# Chimeric Peptides/Proteins Encoded by circRNA: An Update on Mechanisms and Functions in Human Cancers

**DOI:** 10.3389/fonc.2022.781270

**Published:** 2022-02-11

**Authors:** Faiz Ali Khan, Bernard Nsengimana, Nazeer Hussain Khan, Zhenhua Song, Ebenezeri Erasto Ngowi, Yunyun Wang, Weijuan Zhang, Shaoping Ji

**Affiliations:** ^1^ Laboratory of Cell Signal Transduction, Department of Biochemistry and Molecular Biology, School of Basic Medical Sciences, Henan University, Kaifeng, China; ^2^ School of Life Sciences, Henan University, Kaifeng, China; ^3^ Department of Basic Sciences Research, Shaukat Khanum Memorial Cancer Hospital and Research Centre (SKMCH&RC), Lahore, Pakistan

**Keywords:** circRNAs, chimeric peptides/proteins, cancer, biomarkers, therapeutic target

## Abstract

The discovery of circular RNAs and exploration of their biological functions are increasingly attracting attention in cell bio-sciences. Owing to their unique characteristics of being highly conserved, having a relatively longer half-life, and involvement in RNA maturation, transportation, epigenetic regulation, and transcription of genes, it has been accepted that circRNAs play critical roles in the variety of cellular processes. One of the critical importance of these circRNAs is the presence of small open reading frames that enable them to encode peptides/proteins. In particular, these encoded peptides/proteins mediate essential cellular activities such as proliferation, invasion, epithelial–mesenchymal transition, and apoptosis and develop an association with the development and progression of cancers by modulating diverse signaling pathways. In addition, these peptides have potential roles as biomarkers for the prognosis of cancer and are being used as drug targets against tumorigenesis. In the present review, we thoroughly discussed the biogenesis of circRNAs and their functional mechanisms along with a special emphasis on the reported chimeric peptides/proteins encoded by circRNAs. Additionally, this review provides a perspective regarding the opportunities and challenges to the potential use of circRNAs in cancer diagnosis and therapeutic targets in clinics.

## Introduction

**Figure 1:**
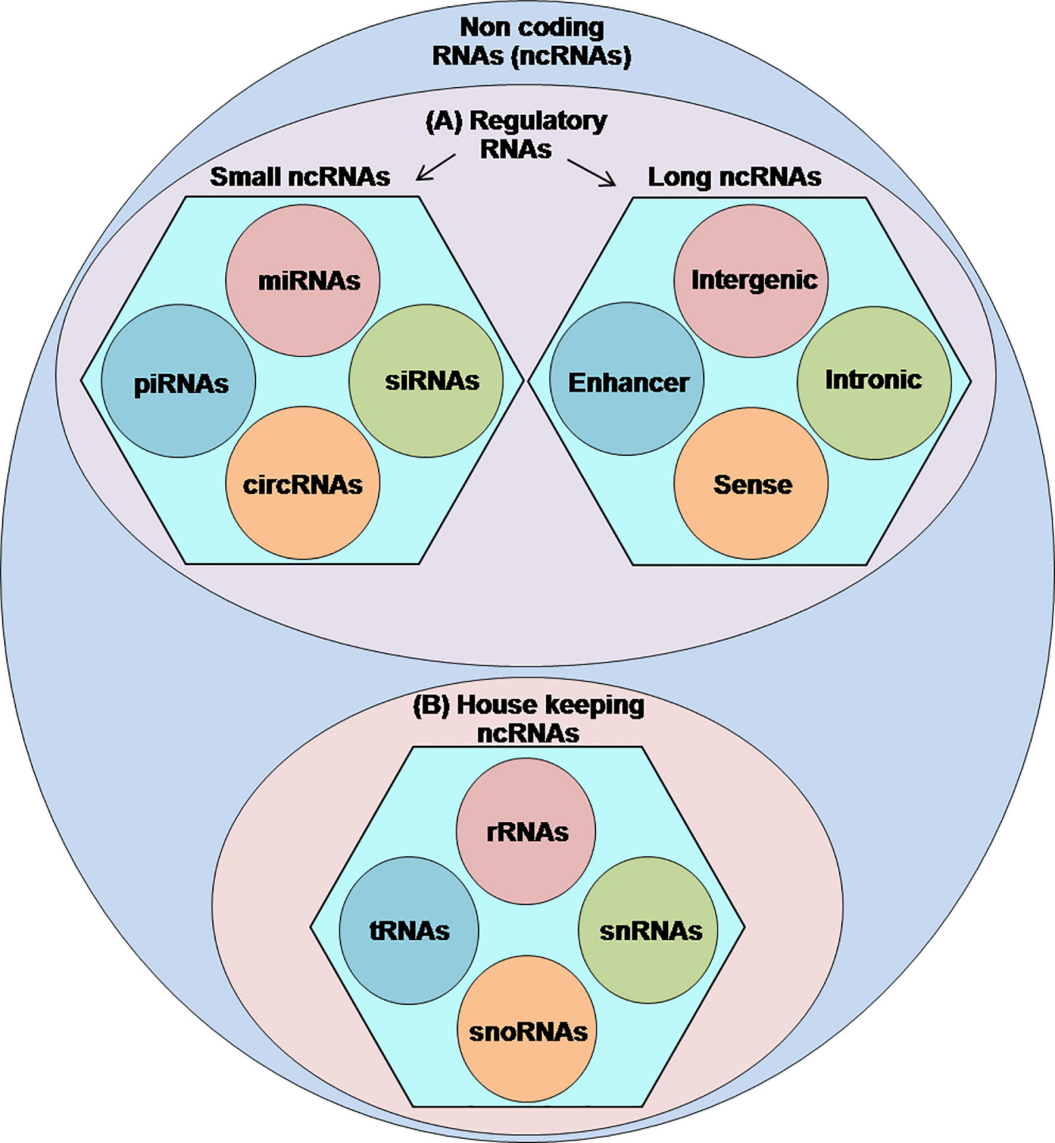
Classification of ncRNAs: a figurative discreption of ncRNAs into **(A)** regulatory ncRNAs and **(B)** housekeeping ncRNAs.

Sequencing of the human genome has indicated that less than 2% of the human genome codes proteins while most of those remaining form the non-coding RNAs (ncRNAs) (1). Therefore, by definition, ncRNAs are transcribed from the genomic DNA but lack the ability to encode proteins (2). Currently, the exact number of ncRNAs within the human genome remains uncertain, however, numerous studies have acknowledged their functional importance in regulating many vital cellular events, including the transcription of their host genes, chromatin modifications, messenger RNA (mRNA) splicing, RNA stability, DNA methylation, and translation (3–8). Classification of ncRNAs into the functional categories is still under different challenges; however, the published literature classifies the ncRNAs into two types: (A) regulatory ncRNAs, which are further subdivided into long ncRNAs (lncRNAs) (>200-nt) and small ncRNAs (<200-nt) consisting of piwi-interacting RNAs (piRNAs), small interfering RNAs (siRNAs), micro RNAs (miRNAs), and circular RNAs (circRNAs) and (B) housekeeper ncRNAs comprising transfer RNAs (tRNAs), ribosomal RNAs (rRNAs), small nuclear RNAs (snRNAs), and small nucleolar RNAs (snoRNAs) (4, 9–11) (**Figure 1**).

Endogenously expressed in the eukaryotic cells, circRNAs are mainly localized in the cytoplasm and characterized as a relatively stable, enriched in exosomes compared to linear RNAs, and highly conserved (4, 12–14). Anatomically, these circRNAs lack the 5ʹ and 3ʹ termini which confer them resistance to exonucleolytic degradation by RNase R (12). Based on their unique properties and functional involvement, literature categorizes the circRNAs into four subtypes: exonic circRNAs (ecircRNAs), which are made of exon(s) (>90% of all circRNAs); circular intronic RNAs (ciRNAs) composed of introns; exonic–intronic circRNAs (EIciRNAs) which contain both intron(s) and exon(s); and intergenic circRNAs composed of fragments of two intronic circRNAs or intergenic sequences (4, 15–17).

Since their discovery back in the 70s, circRNAs and their functional outcomes in the form of encoded peptides/proteins have attracted broad attention. Presently, circRNAs and their encoded peptides/proteins are among the hot topics to be explored for various cellular functions, where they determine the specific functions by regulating the expressions of the host and other genes. Taking part in the fundamental cellular process like gene expression, cell proliferation, and migration of cancer cells, the functional outcomes of these circRNAs in the form of encoded peptides/proteins are considered as a double-edge sword in cancer, where they determine prognosis in different cancers.

Here in this review, we sought to outline the recent advances in the therapeutic study of circRNAs and their peptides/proteins toward cancer therapy and diagnosis. Additionally, this review summarizes the current state of knowledge and future prospective about the use of circRNAs and their peptides/proteins in cancer clinics. We expect that this review could promote the blooming growth of research on circRNAs and their use in the cancer clinic in the near future.

## Biogenesis of circRNAs

**Figure 2:**
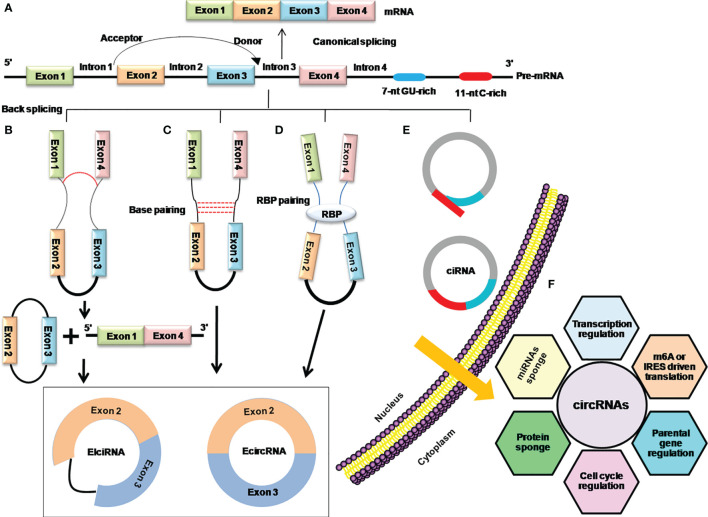
Mechanistic depiction of four circRNA biogenesis models. **(A)** Canonical splicing for the formation of linear mRNA. **(B)** Lariat-driven circularization model of internal splicing, either the removing of introns or retaining in the lariat. **(C)** Intron base-pairing-driven circularization through a base-pairing mechanism in which removing or retaining the introns generates ecircRNA, or EIciRNA especially. **(D)** RBP-driven circularization model in which RBPs shorten the distance between donor and acceptor sites and facilitate removing or retaining the introns to generate ecircRNA or EIciRNA. **(E)** The biosynthesis of stable ciRNA by debranching and exonucleolytic degradation. **(F)** Functions of circRNAs.

Although, there are very limited and incomplete data about the genesis of circRNAs, recent findings have shown that back splicing, intronic complementary sequences (ICSs), and RNA-binding proteins (RBPs) are primary processes in the biogenesis of circRNAs and the hotspots of modern-day research in this domain (12, 18, 19) (**Figure 2**).

Early days’ research on circRNAs found that the phenomena of lariat-driven circularization and intron base-pairing-driven circularization are responsible for the synthesis of circRNAs by catalyzing back splicing and joining the splicing donor and acceptor sites to form the ecircRNAs or EIciRNAs (12, 20). Later on, it was further explored that apart from the back-splicing way, ecircRNAs or EIciRNAs are generated by interactions of RBPs with introns flanking regions that connect the upstream and downstream introns (5). Moreover, the consensus motifs containing GU-rich and C-rich elements in the intronic region can produce the ciRNAs (16) (**Figures 2B–E**). The Quaking (QKI), Muscle blind (MBL/MBLN1), and adenosine deaminase acting on RNA (ADRA1) are among the key regulators of circRNA biogenesis. QKI has been shown to regulate the formation of circRNAs during human EMT by utilizing the QKI-binding domain located in the introns (5), while Muscle blind (MBL/MBLN1) is involved in the circularization of circRNAs in humans *via* its multiple MBL-binding domains in the flanking introns (19). Furthermore, it demonstrates that depletion of the double-strand RNA-editing enzyme–ADRA1 protein, is involved in converting adenosine to inosine on inverted ALU motif, and contributes to the promotion of circRNA production (21, 22).

## Regulatory Roles of circRNAs

As mentioned above, with advancement in high-throughput RNA sequence techniques, and other genomic approaches, circRNAs are being explored for various cellular functions characterized by both tissue-specific and developmental stage-specific manners (23, 24). However, most functions of circRNAs reported are in a developmental stage-specific manner, where they have expression associations with parental genes, thus playing a role in protein coding gene functions.

### Regulatory Roles of circRNAs in Host Gene Expression

Literature mining reveals that circRNAs regulate their host gene expression by epigenetic modulation, transcription, RNA splicing, and translation (8). The association of these circRNAs with parental gene expression has been explored well *via* silencing or depletion of specific circRNAs.

For example, the depletion of ciRNAs ci-mcm5 and ci-sirt7 significantly decreases the host gene expression and alters the cell physiology (25). Similarly, it has been found that depletion of ci-ankrd52 also reduces its parent mRNA expressions by accumulating in the transcription initiation site and by partially co-localizing with the polymerase-II (Pol II) enzyme (25). It has been revealed that the depletion of EIciRNAs circEIF3J and circPAIP2, located in the nucleus, resulted in a significant decrease in expressions of *EIF3J* and *PAIP2* genes, respectively (17). According to Chen and colleagues, ecircRNA: circFECR have associations with the regulation of their parental gene *FLI1* expression, where they interact with its promoter and incorporate *TET1* demethylase to trigger the demethylation in the CpG islands (7). In the same way, circFECR1 also downregulates *DNMT1*, an enzyme that is responsible for sustaining DNA demethylation during DNA replication, thereby regulating *TET1* and *DNMT1*, and their subsequent effects are observed in breast cancer (BC) progression (26). It has been found that, in a condition when circRNA and its parental gene have the same exons, the formation of circRNA will compete with the splicing of pre-mRNA, resulting in low linear mRNA levels (26).

### Regulatory Roles of circRNAs in a Developmental Stage-Specific Manner

Increasing evidence are available on the functions of specific circRNAs in the development stages. Up till now, the vast number of identified circRNA expressions has been well explored in developmental stage-specific manner (27).

Recent findings reveal that circ-mbl, the highly abundant transcriptional product of the Drosophila muscle blind (*mbl*) gene, plays a regulatory role in the developmental process by competing with the linear mRNA production to regulate the expression of the *mbl* transcript (19). circ-sry circRNA consists of a single exon formed from the *Sry* gene and is mainly localized in the cytoplasm. It is abundantly expressed in adult testis and is generally implicated in sex determination (28). Another study has shown that ciRS-7 circRNA derived from the *Cdr1* gene is highly expressed in the mammalian brain and associated with neuronal development (21). In zebrafish, miR-7 expression has an association with its normal development of the midbrain. It has been evaluated that when ciRS-7 is abnormally expressed in zebrafish, defects in the midbrain development are observed and vice versa in the revised case (4). Besides the association with those development-related genes, hsa_circ_2149 has been reported to be distinctively expressed in CD19^+^ but not in the CD34^+^ leukocytes, indicating that it may be involved in the CD19^+^ activities during normal development (4). However, there is a dearth of data on its exact function.

### Regulatory Roles of circRNAs as miRNA Sponges

Along with their developmental stage-specific manner, circRNAs also function as a miRNA sponges. In human, miRNAs directly bind to the target sites primarily within the untranslated region (UTR) of mRNA and might play a vital role in post-transcriptional regulations of gene expression (29). Recently, it has been shown that the regulatory function of miRNAs is affected by the miRNA sponge sites presented in the competent endogenous RNA (ceRNAs) in human (30). The circRNAs are known as natural miRNA sponges due to the presence of multiple miRNA response elements (MRE) on their sequence, which regulates the expression of relevant genes (27).

CDR1as is a circRNA, with >70 conserved binding sites for mir-7 that suppresses its target gene activity. CDR1as is involved in altering miR-7 target gene expression and affects their functions in target cell lines (31). In another study, it has been observed that circTADA2A binds to miR-203a-3p, where it determines the expression of the SOCS3 gene and leads to the attenuation of cell migration, invasion, and clonogenicity in triple-negative breast cancers (TNBC) (8). Testis-specific circSry contains 16 mir-138 target sites. It has been found that it modulates the mir-138 target gene expression and leads to abnormality in cell functioning (32). Furthermore, circRNA produced from *ZNF91* contains >20 sites for miR-23 and miR-296, sequestering these miRNAs’ roles (32). Several other circRNAs that have been found to serve as miRNA sponges including circHRCR (heart-related circRNA) for miR-223 (33), hsa_circ_001569 for miR-148a (34), circITCH (itchy E3 ubiquitin-protein ligase) for miR-214, miR-17, and miR-7 (35), circ-foxo3 (forkhead box protein O3) for miR-29a-3p (36), circHIPK3 (homeodomain-interacting protein kinase 3) for miR-558 (37), circMTO1 (mitochondrial tRNA translation optimization 1) for miR-9 (38), cirZNF609 (zinc finger protein 609) for miR-145 (39), and circBIRC6 (baculoviral IAP repeat-containing 6) for miR-34a and miR-145 (40). Additionally, it was found that the binding to miRNAs may not always lead to inhibition of target genes and the spongy properties of some circRNAs are limited (27).

### Regulatory Role of circRNAs as RBP Sponges

In addition to serving as miRNA sponges, circRNAs also contain binding sites for RBPs and therefore act as protein sponges or decoys (8, 41). RBPs bind to the particular RNA sequences in their target genes and control all cycles of mRNA including splicing, stability, nuclear exportation, and subcellular localization (42). The current research exploration is mainly directed to investigate their potential role in regulating other oncogenes and tumor onset. A study reports that circ-Ccnb1 suppresses mutant *p53* by binding to H2AX and interacts with Ccnb1 and cyclin-dependent kinase 1 (Cdk1) proteins, resulting in the inhibition of tumor progression (43). The upregulation of circACC1 has also been associated with the activation of the AMP-activated kinase (AMPK) pathway in colorectal cancer (CRC) tissues (44). Recently, Chen et al. have shown that ci-AGO2 binds to and interacts with human antigen R (HuR) protein, thereby repressing the functions of the AGO2–miRNA complex and promoting tumorigenesis and aggressiveness (45). Similarly, circPABPN1 can also bind to the HuR protein and inhibit the binding of HuR to PABPN1 mRNA. However, researchers are exploring furthermore about the intrinsic role of circRNAs in these critical molecular processes, expression, and regulation of genes involved in the cell cycle.

## Mechanisms of circRNA Translation Into Peptides/Proteins

Considering the vitality of 5′ and 3′ UTR for the translation initiation in eukaryotic cells, in conventional opinions, lacking of 5′ and 3′ UTRs led researchers to consider circRNAs as ncRNAs for a long period of time. However, later on, studies have accumulated mounting evidence for circRNAs’ protein-coding potential. Sequence analysis demonstrates that there are some circRNAs which comprise the initiation codon AUG and stop codons followed by putative open reading frames (ORFs) with commendable length, which provides the circRNAs potential of translation (46–50).

There are explanations of circRNA translations *via* canonical cap-dependent translation in which a 7-methylguanosine cap is added to the 5′ end of mRNA for translation initiation. This initiation is mediated and guided by the unique set of eIF4E translation initiation factors, a protein complex including eIF4E, eIF4G, and eIF4A components (51–53). Another possible way of circRNA translations is cap-independent translation. It is a naturally occurring alternative mechanism employed in eukaryotes under certain conditions, such as cellular stress, viral infection, physiological stimulation in cell differentiation, and synapse network formation. In this mode of translation, a special sequence located in the 5′ UTR of mRNAs, such as an internal ribosome entry site (IRES), induces translation initiation (54, 55).

More recently, it has been found that majority of circRNAs originate from protein-coding genes containing IRES-like elements and N6-methyladenosine (m^6^A) modifications that initiate translation, suggesting that circRNAs can be translated into peptide/proteins (56). It has been categorized by two potential models which determine the regulation of circRNA translation initiation. In the first mechanism for circRNA translation initiation, coding circRNAs containing natural IRES-like elements can be directly recognized by a non-canonical eIF4G protein (eIF4G2 or DAP-5) and regulate the circRNA translation initiation (57). eIF4G2 contains the eIF4A- and eIF3-binding sites but lacks the eIF4E-binding region. Thus, in IRES-initiated translation, IRES can assemble the eIF4 complex in the absence of eIF4E, directly initiating translation (58–62). These findings were further endorsed by a study by Chen and Sarnow in which they found that engineered circRNAs in artificial constructs containing IRES-like elements could recruit the 40s ribosomal subunit, initiate translation, and produce a long repeating polypeptide chain with continuous ORFs in an *in-vitro* experiment (63). Another study has further confirmed that functional proteins can be translated from circRNAs by inserting the IRES-like elements into a minigene containing split green fluorescent proteins (GFP) (64). Similarly, Perriman and Ares show that artificial circular mRNA in *Escherichia coli* can be translated into long protein chains containing ORF for GFP, implying that bacterial ribosome can repeatedly induce the expression of GFP from circular mRNA (65) and yield a 795-nt-long circular mRNA containing ORF of 30-kDa peptide, representing that the bacterial ribosomes scanned the circular mRNA more than ten times. In summary, the studies above demonstrate that circRNAs can function as messenger RNA and be translated into proteins *via* IRES-like regions that are independent of 5ʹ cap and 3ʹ poly (A) tail.

The other important cap-independent translation mechanism is through methylated adenosine residues in the form of m^6^A in the 5′ UTR (66, 67). m^6^A is the common modification of mRNA, regulates many cellular events, such as tissue development, DNA damage response, and sex determination, and is also involved in tumorigenesis (68–70). Numerous translatable endogenous circRNAs enriched with consensus m^6^A motifs can promote efficient initiation of translation. Mechanistically, the m^6^A reading protein YTHDF3 (YTH domain family protein 3) recognizes m^6^A and recruits eIF4G2 to m^6^A, where eIF4G2 recognizes the IRES and initiates the assembly of the eIF4 complex, leading to the initiation of translation. Furthermore, m^6^A-initiated translation is cell-type independent and a single m^6^A site is sufficient to bypass the m7G cap recruitment and initiate the cap-independent model of translation (64, 71).

To date, some endogenous circRNAs have been linked with polysomes, implying that a considerable number of these molecules are able to be translated into peptides/proteins (64, 72, 73). Both IRESs and m^6^A-mediated translation initiation are considered as the most prevalent and essential components of circRNA translation initiation. Alternatively, rolling circle amplification (RCA) has been anticipated as another putative mechanism of circRNA translation (46, 74). Abe et al. have reported that circRNAs can efficiently be translated into peptides/proteins by a RCA mechanism with an infinite ORF either in a cell-free rabbit reticulocyte lysate system or in living human cells (46), suggesting that apart from IRESs and m^6^A, there are other elements that can initiate cap-independent translation within the circularized transcripts (46, 75). Although the above-listed mechanisms for circRNA translation provide enough explanation, researchers are still looking for further exploration to connect more dots.

In literature, numerous methods have been listed for the identification of functional peptides/proteins encoded by circRNAs including the prediction of ORFs, IRES-like elements, m^6^A modification, conservation analysis, translation omics, proteomics, and experimental analysis (76).

Considering the rapid developments in the protein-coding circRNA field, the foremost aims of this review is to expand our current understanding of the protein-coding ability of circRNAs and the function of the peptides/proteins especially in cancer.

## Peptides/Proteins Encoded by circRNAs

According to their effects and functions, circRNA-encoded peptides/proteins can be divided into four types: non-cancerous regulatory, promoting and inhibiting their full-length proteins, and signal transducer mediators. As per the study outlines, those peptides generated by circRNAs and having regulatory functions in other physiological processes except cancer are designated as non-cancerous regulatory peptides/proteins (61). The roles of circRNA-encoding peptides/proteins are up to the characteristic of overlapping amino acid sequence with their cognate linearly spliced protein isoforms, so it can act as a decoy to enhance their cognate linearly spliced protein isoform functions by releasing the proteins or protecting them from degradation, enhancing the expression of relevant proteins (77). Opposite to their role in enhancing the expression of relevant genes, there are other classes of circRNA-encoded peptides/proteins, which play an inhibitory role in reducing the activation of their cognate linearly spliced proteins (78). In the fourth category of translated peptides, there are some peptides taking part in signal transduction pathways to affect downstream targets but have no clear association with their full-length proteins (79).

**Figure 3:**
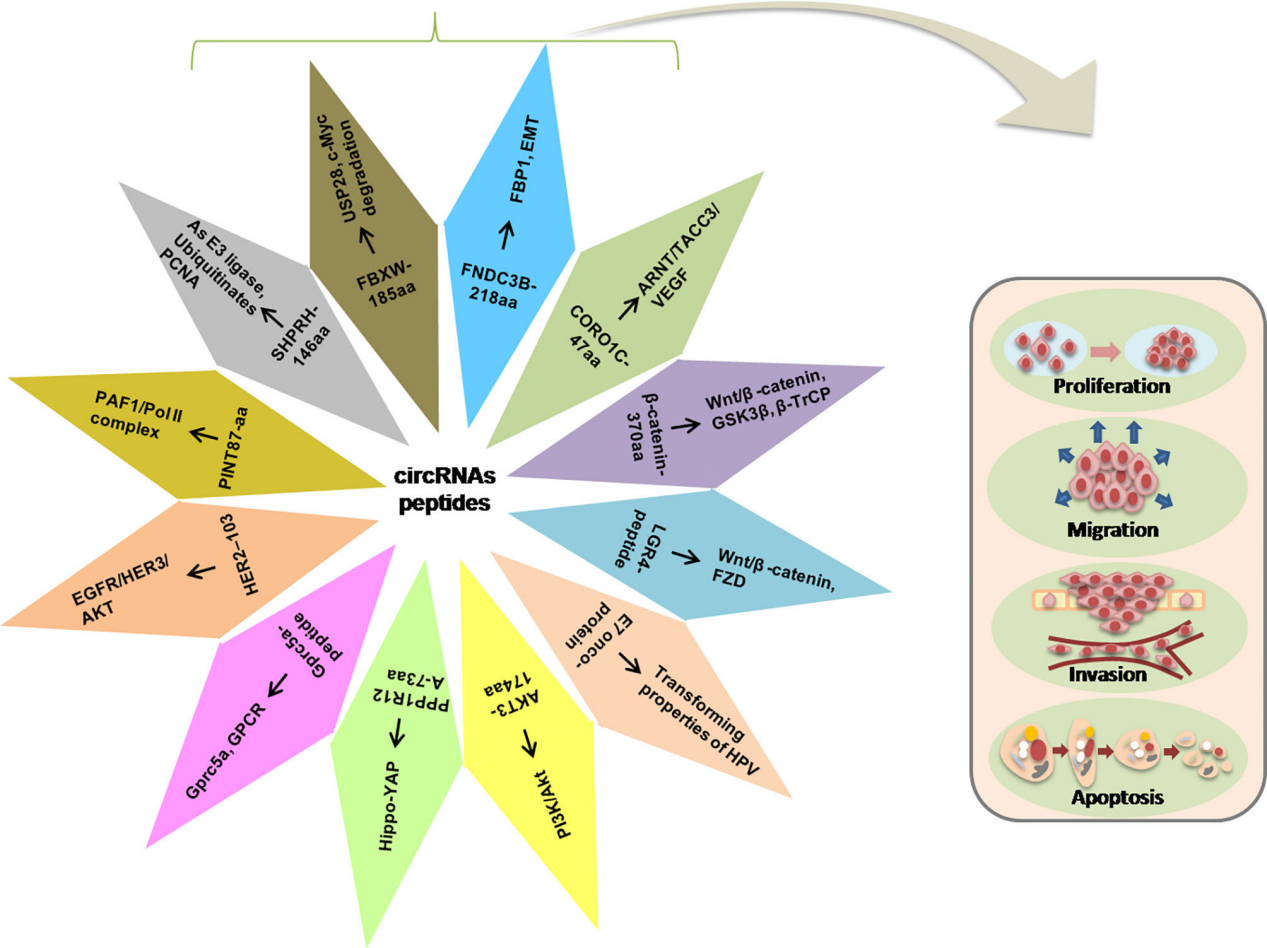
Illustration of the circRNA-encoded peptides/proteins and signaling pathways in the progression of tumorigenesis.

Herein, we discussed some functional peptides/proteins encoded by the circRNAs and their possible mechanisms involved in regulating the various cellular activities such as proliferation, metastasis, and invasion (**Figure 3**).

## Non-cancerous Regulatory Peptides/Proteins

### Translation of circMbl Regulates Neuronal Function

In the functional peptides encoded by circRNAs, circMbl is the firstly discovered peptide. circMbl originates from the second exon of *mbl* and regulates its parental gene expression by competing for the start codon with its canonical linear mRNA (19). Later on, it has been further detected that circMbl encodes a protein by using the cUTRs (UTR from circMbl) that act as IRES-like elements in a FOXO-dependent manner (61). The presence of MBL-binding sites in the flanking intronic sequences of the *mbl* gene promotes circMbl production from its exons. circMbl is present in neural related genes in the fly heads, providing a link between RBPs and brain functions. In addition, circMbl and their putative encoded isoforms are present in synaptosome fractions and regulate the synaptic functions induced by acute fasting through 4E-BP and FOXO (61). Despite the absence of signal peptide sequence detection, research reveals that circMbl is translated in the synapses, where they associate with RBPs and mediate the different roles in neurodegenerative disorders (80). However, to date, there is no available evidence on their involvement in tumorigenesis.

### Translation of circ-ZNF609 Regulates Myoblast Proliferation

circ-ZNF609 (hsa_circ_0000615) is highly conserved, containing 753-nt ORF derived from the second exon circularization of its parental gene in muscle cells (81). The analysis of the protein-encoding ability of circ-ZNF609 using artificial vector P-circ3XF reveals that its UTR can drive IRES-dependent translation and encode a novel peptide isoform in a splicing-dependent manner, and mediates the regulation of myoblast proliferation (81). *FOXP4* (Forkhead box P4) majorly reported in many cancers, is also regulated by circ-ZNF609 through sponging mir-138-5p in renal carcinoma, resulting in the downregulation of cell proliferation and invasion processes (82). Furthermore, circ-ZNF609 promotes the expression of p70S6K by binding to and sequestering miR-145-5p, thus elevating BC progression (39). Additionally, highly expressed circ-ZNF609 binds to and inhibits the activity of miRNA-150-5p, therefore upregulating the Sp1 expression and facilitating the metastasis of nasopharyngeal carcinoma cells (83). According to the recent findings, it has been evaluated that circ-ZNF609 is an important regulator of G1/S transition circ-ZNF609 knockdown reduces the phosphorylated Rb : Rb ratio and E2F1 expression, which leads to cell cycle arrest at the G1/S transition. Conversely, the increased circ-ZNF609 expression in rhabdomyosarcoma promotes cell proliferation (84). Taken together, it can be concluded that Circ-ZNF609 is upregulated in different cancers, and its downregulation can inhibit cancer progression.

## Promoting the Function of Full-length Proteins

### Translation of circ-FBXW7 in Glioblastoma and TNBC

It has been demonstrated that circ-FBXW7 (hsa_circ_0001451) is formed by the exon 3 and exon 4 circularization of the *FBXW7* gene in glioblastoma samples. circ-FBXW7 has to span the back-splice junction ORF of 620-nt encoding a 22-kDa novel protein driven by an IRES in a 5ʹ cap-independent translation manner, termed as FBXW7-185aa (77). The expression of circ-FBXW7 is positively associated with the overall survival of glioblastoma patients. It has been found that in glioblastoma patients, the overexpression of FBXW7-185aa inhibits the proliferation of cancer cells *in vivo* and *in vitro* (56). Furthermore, an isoform of the *FBXW7* gene FBXW7α is also reported to target c-Myc for ubiquitination-induced degradation (85). Mechanistically, the de-ubiquitinating enzyme ubiquitin-specific protease 28 (USP28) binds to and stabilizes c-Myc by interacting with the N-terminus of FBXW7α. However, due to its high affinity, FBXW7-185aa binds to and inhibits USP28, thereby freeing FBXW7α to degrade c-Myc in cancer cells (77). The expression of circFBXW7 in TNBC is associated with poor clinical outcomes (86), whereas its upregulation suppresses TNBC progression by binding to the tumor-promoting miRNA (miR-197-3p) (87). circFBXW7 encodes the same protein (FBXW7-185aa) to repress malignant progression. FBXW7-185aa inhibits the proliferation and migration of TNBC cells by inducing *c-Myc* degradation (87). Based on this experimental evidence, it is reasonable to consider that circ-FBXW7 and FBXW7-185aa play an important role in glioblastoma and TNBC prognosis. Therefore, these might serve as independent prognostic, therapeutic markers in glioblastoma and TNBC.

### Translation of circ-SHPRH in Glioblastoma and Hepatocellular Carcinoma (HCC)

circ-SHPRH (hsa_circ_0001649) is formed after the back splicing of exons 26–29 of the SNF2 histone linker PHD RING helicase (*SHPRH*) gene and contains only 440-nt. The circularization of circ-SHPRH results in a tandem stop codon “UGAUGA”, starting the translation initiation and termination through the overlapping codon (88). circ-SHPRH encodes a 17-kDa protein named “SHPRH-146aa” formed by spinning the back-splice junction ORF and driven by IRES-like elements (88). Both circ-SHPRH and its transcripts are highly expressed in normal human brain cells, participating in the inhibition of central nervous system cancers through the ubiquitin-proteasome pathways triggered by SHPRH (88, 89). Recently, it has been found that the overexpression of SHPRH-146aa reduces the malignancy of glioblastoma cells with increased levels of SHPRH-146aa, and glioblastoma patients have increased survival time. Mechanistically, SHPRH-146aa acts as a protective decoy molecule to protect SHPRH from degradation by the ubiquitin-proteasome. Full-length SHPRH ubiquitinates proliferating cell nuclear antigen (PCNA) as an E3 ligase, resulting in cell proliferation repression (56).

Moreover, circ-SHPRH harbors potential binding sites for RBPs (U2AF65, EIF4A3, UPF1), and miRNAs (miR-1283, miR-4310, miR-182-3p, miR-888-3p, miR-4502, miR-6811-5p, miR-6511b-5p, and miR-1972), and may act as a sponge and regulate the progression of HCC (90).

### Translation of circE7 in Cervical Carcinoma

circE7, a 472-nt circRNA, contains the entire E7 ORF, originated *via* back splicing by human papillomaviruses (HPV). circE7 with modification of m^6^A is mainly localized in the cytoplasm, bearing polyribosome, and encodes the E7 oncoprotein (91). circE7 is predicted to have binding sites for multiple miRNAs, but none of these binding sites is conserved among HPV species. Cell stressors, such as heat shock (42°C), upregulate the E7 protein expression by a two- to four-fold increase (91). In addition, the depletion of circE7 in cervical carcinoma cells reduces E7 protein levels and inhibits cancer cell growth *in vitro* and *in vivo*. The circE7 knockdown by shRNA recovered circResist_WT but not circResist_noATG, confirming that the protein-coding ability of circE7 is required for its function *in vivo*. The above results provide the first evidence that protein-encoding circRNAs derived from a virus are biologically functional and link to the transforming properties of some HPV.

Although the circ-E7 and E7 peptides play an important role in the ability of HPV to transform cervical cells, the exact mechanism underlying the activity of the E7 peptide is unknown, and more studies are needed to understand the mechanism through which the E7 peptide exerts its function.

### Translation of circß-catenin in HCC

circ-0004194 (termed as circβ-catenin) is derived through back splicing of six exons of the *β-catenin* gene containing 1,129-nt. Subsequent analysis shows that circβ-catenin is translated into a novel 370aa (40.8-kDa) peptide designated as β-catenin-370aa, mainly localized in the cytoplasm and positively correlated with its linear mRNA isoform in humans (92). circβ-catenin regulates the β-catenin expression at the protein level rather than its transcription level. circβ-catenin is significantly expressed in both HCC tissues and cell lines. The construction of the circβ-catenin knockdown expression vector inhibits the full-length β-catenin and β-catenin-370aa protein levels without affecting the β-catenin mRNA. Knockdown of circβ-catenin suppresses HCC cell growth and metastasis through the inhibition of Wnt/β-catenin pathway. The stability of β-catenin is strongly associated with its phosphorylation status. Glycogen synthase kinase 3 beta (GSK3β) interacts with and phosphorylates β-catenin, and the ubiquitin ligase β-TrCP ubiquitinates the β-catenin, leading to degradation by the proteasome (93). Therefore, it is evaluated that β-catenin-370aa binds to GSK3β, which eventually leads to prevent the GSK3B from interacting with full-length β-catenin (92, 94).

Overviewing the four different forms of peptides which regulate the expression of genes in different cancers, it could be speculated that they may act as targets of drug designing for cancer treatment.

## Inhibiting the Function of Full-length Protein

### Translation of circ-AKT3 in Glioblastoma

circ-AKT3 (hsa_circ_0017250) is generated from circularization of exons 3–7 of the parent gene with 524-nt, localized in the cytoplasm (78). circ-AKT3 encodes a 174aa peptide (AKT3-174aa) through the overlapping start–stop codon UAAUGA driven by active IRES and contains a similar “aa” sequence to AKT3 residues from positions 62 to 232 (78).

AKT3-174aa expression is lowered in glioblastoma cells and is negatively associated with the diagnosis of the disease, suggesting that it may be a potential biomarker (78). In addition, the overexpression of AKT3-174aa diminishes glioblastoma cell proliferation, tumor growth, and radiation resistance, while its knockdown increases the malignant phenotypes, indicating that AKT3-174aa, but not circRNA itself, exerts a tumor-inhibiting role in glioblastoma cells (78). PI3K/Akt performs a key function in multiple oncogenic signaling pathways that drive the development and progression of glioblastoma (95). The activated PI3K recruits AKT to the plasma membrane through the PH domain and sequentially phosphorylates AKT at thr308 and ser473, respectively, to undergo a successive activation. Pyruvate dehydrogenase kinase isozyme 1 (PDK1) (another PH domain-containing kinase) directly phosphorylates AKT at thr308, the most critical step for AKT activation. The resemblance of the sequences between AKT3-174aa and AKT3 infers that AKT3-174aa can compete and bind to PDK1, thereby reducing the level of AKT3 thr308 phosphorylation, and executes a negative regulatory role in the PI3K/AKT signaling pathway (78). Another circRNA, circAKT3 (hsa_circ_0000199), originated from exons 8–11 of the AKT3 gene (96). It is highly upregulated and associated with an aggressive phenotype in gastric cancer (GC) patients with resistance to cisplatin (CDDP) therapy. circAKT3 enhances DNA damage repair, inhibits the apoptosis of GC cells, and promotes PIK3R1 expression by sponging miR-198 through PI3K/AKT pathway activation in GC (96). Thus, the above data show that circAKT3 might be a potential therapeutic biomarker for GC patients receiving CDDP therapy (96).

## Signal Transducer Peptides/Proteins

### Translation of circPINTexon2 in Glioblastoma

Produced by self-circularization of exon 2 of the lncRNA LINC-PINT, circPINT (hsa_circ_0082389) contains 3ʹ AG and 5ʹ GT sequences required for back-splicing. circPINTexon2 has been determined endogenously in human cell lines, and its corresponding up-or downregulation has been confirmed by the artificial overexpression or junction siRNA transfection. It contains the ORF and IRES-like elements that initiate translation through 5ʹ cap-independent translation machinery to encode a 10-kDa protein designated as 87aa peptide (79). Both circPINTexon2 and its peptide “PINT87aa” are downregulated in glioblastoma tissue, with PINT87aa having a negative impact on clinical prognosis. PINT87aa localized in the nucleus, which plays a tumor-suppressive role in the control of cell proliferation and tumorigenesis. PINT87aa can interact with the PAF1 complex, inhibiting the transcriptional elongation of multiple oncogenes (50).

The polymerase-associated factor (PAF1) complex is an important component in RNA polymerase II, regulating gene transcription and elongation. It has been shown that the PINT87aa peptide interacts with PAF1, keeps the PAF1 complex on the target gene promoter, and regulates the formation of the PAF1/Pol II complex, thus controlling its downstream effects in the progression of several cancer types, including glioblastoma, BC, HCC, and GC (79). Loss of PINT87aa makes PAF1 lose its proper position, and the freed PAF1 is sequentially involved in many other biological processes such as cell cycle regulation, histone modification, and self-renewal of cancer stem cells (79).

### Translation of circGprc5a in Bladder Cancer

circGprc5a (hsa_circ_02838) is commonly upregulated in cancer stem cells (CSCs) and advanced and metastatic bladder tumors with bad prognosis. It encodes a polypeptide of 11aa termed circGprc5a-peptide, which exerts its function through a peptide-dependent manner (97). Knockdown of circGpr5a and/or its peptide expression diminishes the proliferation and metastasis of bladder CSCs. In regulating self-renewal and metastasis of CSCs, circGprc5a-peptide binds to Gprc5a surface protein and activates the G protein-coupled receptor (GPCR) signaling pathway involved in tumorigenesis (97, 98). Researchers have established circ-Gprc5a*-*mutant cells that cannot produce peptides, which in turn impair the function of circ-Gprc5a. These findings suggest that circGprc5a-peptide promote bladder tumorigenesis and metastasis.

### Translation of circPPP1R12A in Colon Cancer (CC)

circPPP1R12A (hsa_circ_0000423) formed by back splicing of exon 24/25 of the *PPP1R12A* gene located at 12q21.2 contains a 216-nt small ORF that encodes a conserved protein of 10 kDa (circPPP1R12A-73aa) localized in the cytoplasm (99). circPPP1R12A is significantly upregulated in CC, and patients with highly expressed circRNA have a relatively shorter overall survival. It has been demonstrated that mutation of the start codon disrupts circPPP1R12A protein-encoding ability and represses CC proliferation, migration, and invasion both *in vitro* and *in vivo*, indicating that circPPP1R12A-73aa is involved in CC progression. Furthermore, the Hippo-Yes-associated protein (YAP), one of the most conserved tumor-repressor signaling pathways, can also be activated by circPPP1R12A-73aa resulting in the promotion of proliferation, migration, and invasion capability of cancer cells (99, 100). In addition, YAP-specific inhibitor peptide 17 has significantly reduced the proliferation, migration, and invasion abilities of CC promoted by upregulating circPPP1R12A-73aa. Collectively, a protein encoded by circRNA circPPP1R12A contributes to cell proliferation and provides an insight for the development of a novel therapeutic biomarker for CC patients.

### Translation of circLgr4 in CRC

circLgr4 (hsa_circ_02276) is strongly expressed in advanced (metastatic) CRC with a poor prognosis. circLgr4 is translated into a 19aa (3-kDa) peptide, which plays an essential role in CSCs maintenance, renewal, and invasion (101).

A Wnt/β-catenin signaling pathway is involved in colorectal CSCs tumorigenesis and metastasis (102). circLgr4-peptide interacts with Lgr4 to promote the activation of Wnt/β-catenin signaling through the ubiquitination of the Frizzled receptors (FZD), resulting in colorectal CSCs self-renewal and invasion. Both circLgr4 ASO (antisense oligo) and Lgr4 ASO can inhibit tumor proliferation (101). The CircLgr4-peptide-Lgr4 axis might be used as a therapeutic biomarker for colorectal CSCs and colorectal tumorigenesis.

### Translation of circFNDC3B in GC and CC

circFNDC3B (hsa_circ_0006156) is structured through back splicing and circularization of exons 5–6 of the fibronectin type III domain-containing protein 3B (*FNDC3B*) gene. circFNDC3B possesses a potential IRES and an ORF of 218aa and encodes a 25-kDa peptide. circFNDC3B promotes cell migration and invasion in GC and is associated with EMT (103). EMT is a complex transformation process that drives epithelial carcinoma cells into malignant phenotype and enables tumor cells to migrate from the tumor’s primary site to distal metastasis (104). The downregulation of E-cadherin (epidermal phenotype protein E) and the upregulation of N-cadherin (mesenchymal phenotype N), Vimentin, and Snail enhance EMT in GC (105, 106). circFNDC3B promotes cell migration and invasion by inhibiting E-cadherin protein expression. circFNDC3B interacts with *IGF2BP3* and promotes CD44 mRNA expression by forming a ternary complex of circFNDC3B–*IGF2BP3–*CD44 mRNA. However, *FNDC3B* has multiple domains which have been explored in cell adhesion, morphology, migration, and embryonic differentiation (107). While working on CC, Pan et al. evaluated that Circ-FNDC3B expression is associated with proliferation, invasion, and migration in CC. Mechanistically, circ-FNDC3B-218aa inhibits the expression of Snail and subsequently promotes the tumor-repressive effect of FBP1, which results in the suppression of tumor progression and EMT. Thus, the novel circ-FNDC3B-218aa might serve as a potential therapeutic target in CC (108). Further studies are required to elucidate the functional/regulatory role of peptides encoded by circFNDC3B in tumorigenesis.

### Translation of circHER2 in TNBC

The novel circRNA of 103aa-long peptide (known as HER2-103) is formed from the circularization of exons 3–7 of the human epidermal growth factor receptor 2 (*HER2*) gene in TNBC samples. It has been further evaluated that patients positive with circHER2/HER2-103 harbored a worse overall prognosis than circHER2/HER2-103-negative patients. HER2-103 promotes malignant phenotypes by interacting and activating the epidermal growth factor receptor (EGFR)/HER3 and promoting AKT phosphorylation (109). Further exploration is needed for its detailed functioning.

### Translation of circ-0000437 in Endometrial Cancer

A solo study has been reported on CORO1C47aa. It originates from the circularization of exons 7–8 of its host gene *CORO1C* and has significantly reduced expression in endometrial cancer compared to matched precancerous tissue. Circ-0000437 contains a short ORF encoding a functional peptide named CORO1C47aa, and its overexpression mediates the inhibition of angiogenesis by suppressing endothelial cell proliferation, migration, and differentiation through competition with transcription factor TACC3 and suppresses VEGF signaling. Results also show that CORO1C47aa is directly bound to ARNT through the PAS-B domain and involved in blocking the association between ARNT and TACC3, which led to a reduced expression of VEGF and angiogenesis. The antitumor effects of CORO1C-47aa on endometrial cancer progression suggest that CORO1C-47aa has a potential role in anti-carcinoma treatment (110).

It is proposed that, in the near future, the ectopic expression and clinical association of these peptides in cancers will be a part of experimental and clinical examination, such as examination of body fluids or immunohistochemistry (IHC) analysis of tumor tissues (76). Regarding this proposal, many novel studies have approved that some peptide-related drugs such as mifamurtide (111), tumor necrosis factor-alpha (TNF-α), interferon-γ (INF- γ) (112), and interleukin-2 (IL-2) (113) cure human diseases including cancer and are found to be more effective (114). Compared to other traditional drugs, these peptide-related drugs hold distinctive advantages, including being highly specific and active, being less toxic, and having low immunogenicity (115, 116).

It has been also approved that some circRNA-encoded peptides/proteins (FBXW7-185aa and SHPRH-146aa) play a role in tumor inhibition. Approaches are now in process to rescue or strengthen these tumor-inhibiting peptides/proteins, including vaccination with artificially synthesized peptides or viral vector vaccines that encode the specific peptide sequences for cancer therapies (117–119). Additionally, these peptides modulate tumor energy metabolism, oncoprotein stability, and the EMT of cancer cells (11, 120).

A summary of recently reported studies on the altered expression of peptides encoded by circRNAs as potential biomarkers for different malignancies is listed in **Table 1**.

**Table 1:** Tumor-related circRNA-encoding peptides/proteins and their functions.

Name of circRNAs	Peptide	Disease	Expression in disease	Murine model used	Cohort (cases)	Mechanism of action	Function	**Reference**
Non-cancerous regulatory peptides/proteins								
circMbl	10-kDa	Unknown	Unknown	*In*-*vitro*	–	Unknown	Regulate neuronal functions	(61)
circZNF609	–	DMD	–	*In*-*vitro*	–	Unknown	Regulate myoblast Proliferation	(81)
Promoting the function of full-length proteins								
circFBXW7	FBXW7-185aa	Glioblastoma	Down	*In*-*vitro*, *in*-*vivo* (nude mice)	38	Enhance degradation of c-Myc caused by FBXW7α	Inhibit proliferation and cell cycle progression in cancer cells	(77)
		TNBC	Down	*In*-*vitro*, *in*-*vivo* (nude mice)	473		Inhibit the proliferation and migration of TNBC cells	(87)
circ-SHPRH	SHPRH-146aa	Glioblastoma	Down	*In*-*vitro*, *in*-*vivo* (nude mice)	60	Enhance ubiquitination of PCNA by inhibiting SHPRH degradation	Inhibit the malignant behavior and tumorigenicity of glioblastoma cancer	(88)
circE7	E7 oncoprotein	Cervical carcinoma	Up	*In*-*vitro*, *in*-*vivo* (NSG mice)	108	Increase the E7 oncoprotein level	Promote the growth and transformation of cervical carcinoma cells	(91)
circβ-catenin	β-catenin-370aa	HCC	Up	*In*-*vitro*, *in*-*vivo* (nude mice)	50	Stabilize β-catenin by antagonizing GSK3β-induced degradation, and activate Wnt/β-catenin pathway	Promote tumorigenesis and metastasis of HCC	(92)
Inhibiting the function of full-length protein								
circAKT3	AKT3-174aa	Glioblastoma (GBM)	Down	*In*-*vitro*, *in*-*vivo* (nude mice)	38	Reduce the phosphorylation of AKT3 thr308	Inhibit cell proliferation, radio resistance and tumorigenicity of GBM	(78)
Signal transducer peptides/proteins								
circPINT	PINT87aa	Glioblastoma	Down	*In*-*vitro*, *in*-*vivo* (nude mice)	38	Enhance PAF1 and its target genes’ promoter interaction and control its downstream effects	Induce cell proliferation and invasion; Inhibit the transcriptional elongation of oncogenes	(79)
circGprc5a	circGprc5a-peptide (11aa)	Bladder cancer	Up	*In*-*vitro*, *in*-*vivo* (mice)	60	Bind to Gprc5A and activate GPCR signaling pathway	Promote metastasis and the self-renewal of bladder CSCs	(97)
circPPP1R12A	circPPP1R12A-73aa	CC	Up	*In*-*vitro*, *in*-*vivo* (nude mice)	120	Activate Hippo-YAP signaling pathway	Promote pathogenesis and metastasis of CC	(99)
circLgr4	circLgr4- peptide	CRC	Up	*In*-*vitro*, *in*-*vivo* (nude mice)	60	Interact with Lgr4 and activate Wnt/β-catenin signaling pathway	Promote the CRC-CSC self-renewal, tumorigenesis, and invasion	(101)
circFNDC3B	25-kDa peptide	GC	–	–	–		Unknown	(103)
		CC	Down	*In*-*vitro*, *in*-*vivo* (nude mice	87	Inhibited the expression of Snail	Inhibit the proliferation, invasion and migration of CC cells	(108)
circHER2	HER2-103	TNBC	Up	*In*-*vitro*, *in*-*vivo* (nude mice)	59	Promote homo/hetero-dimerization of EGFR/HER3 and AKT phosphorylation	Promote cell proliferation, migration and invasion of TNBC cells	(109)
circ-0000437	CORO1C47aa	EC	Down	*In*-*vitro*, *in*-*vivo* (nude mice)	198	Inhibit the VEGF *via* binding to TACC3 or block the association of ARNT and TACC3	Inhibit angiogenesis by suppressing cell proliferation, migration and differentiation of EC cells	(110)

DMD, Duchenne muscular dystrophy; BC, breast cancer; TNBC, triple negative BC; CSC, cancer stem cell; CC, colon cancer; GBM, glioblastoma multiform; GC, gastric cancer; CRC, colorectal cancer; HCC, hepatocellular carcinoma; EC, endometrial cancer.

## circRNA-Encoded peptides/Proteins as Therapeutic Target/Biomarkers

Owing to the distinctive back-splice junction features, high stability, and specificity in tissues and body fluids, circRNAs and their encoded peptides/proteins are considered as therapeutic targets/biomarkers in cancer studies (121–123).

**Figure 4:**
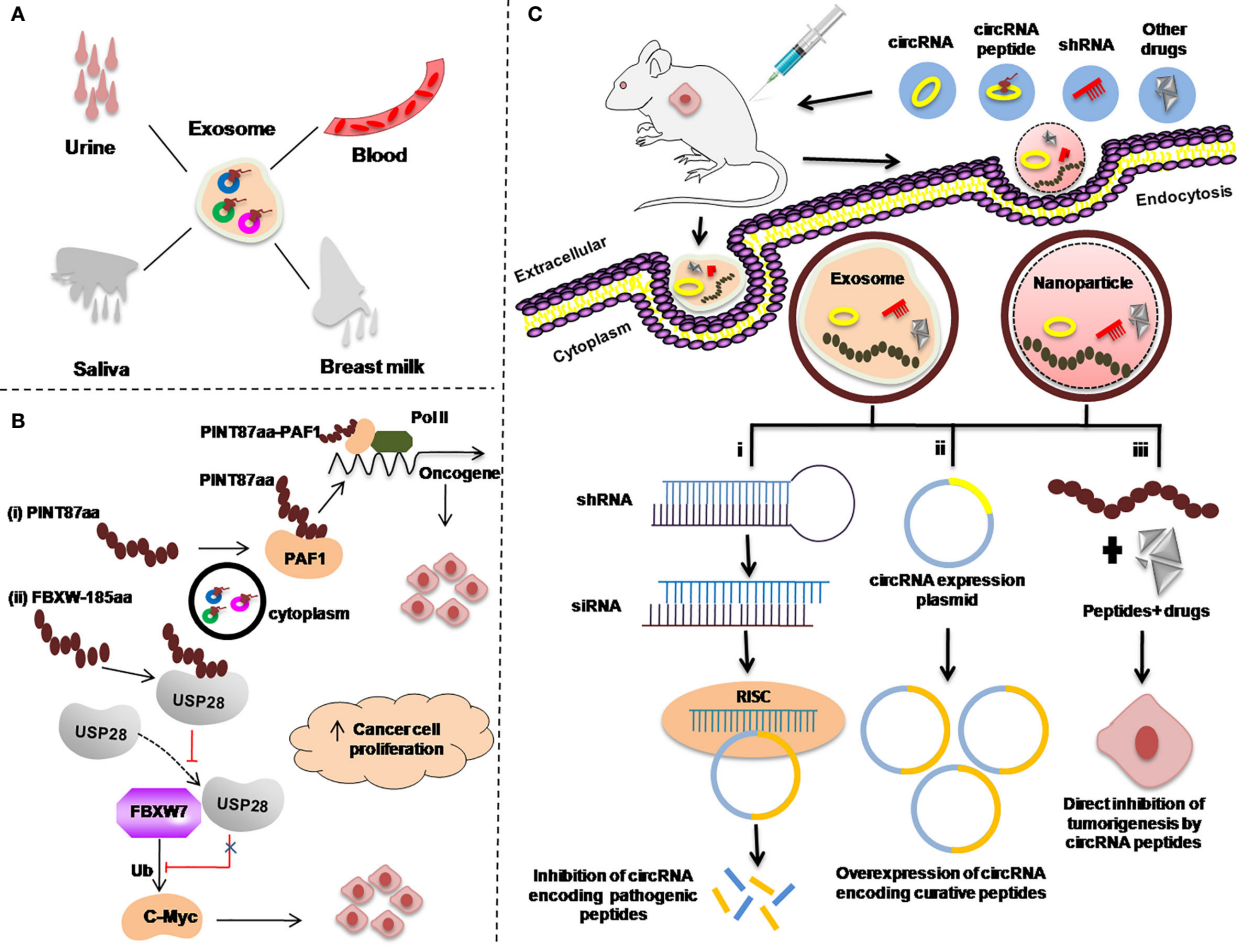
Re-proposed possible applications of circRNA-encoded peptides/proteins in cancers as diagnostic or prognostic biomarkers. **(A)** Body fluids, such as urine, blood, saliva, and breast milk as a sample for examining exosome-circRNA-encoded peptides/proteins for cancer diagnosis. **(B)** Regulation of the expression of circRNA-encoded peptides/proteins to inhibit transcriptional elongation of oncogenes**. (C)** Strategies used to target circRNA peptides as a therapeutic approach in animal mouse models. Exosome-mediated delivery of small hairpin RNA (shRNA) **(i)**. circRNA expression plasmid to overexpress circRNA-encoding curative peptides/proteins **(ii).** Gold nanoparticle-mediated delivery of shRNA targeting the back-splice junction of circRNAs for treating different malignancies **(i–iii)**.

Several techniques have been developed to study/target circRNAs and their encoded peptides/proteins for therapeutic purposes *in vivo*. Here we proposed some possible strategies that could be operational via over-expressing or knock down the circRNA encoding peptides, as summarized in **Figure 4**.

Studies have demonstrated that circRNAs in the exosome and other body liquids such as in blood, urine, saliva, and breast milk have provided a new direction for the uncovering of new biomarkers for cancer diagnosis (124–126) (**Figure 4A**).

Secondly, as it has been reported in many studies that there are several circRNA-encoded peptides/proteins such as PINT87A and FBXW7-185aa which are involved in regulating the expression of different oncogenes (77, 79). Possible strategies to use these peptides/proteins as targets to control the expression of those oncogenic proteins, is to design the corresponding target drugs as summerized in **Figures 4B, C**.

In cancer treatment, knockdown of the circRNAs encoding pathogenic peptides/proteins is the most efficient way to develop circRNA-based therapies (127) [**Figure 4C(i)**].

There are various circRNAs that encode curative peptides/proteins and are downregulated in various malignancies and can be exploited as potential targets for cancer treatment (88, 92). A proposed strategy for these curative peptides/proteins against cancer is explained in **Figure 4C(ii)**.

In addition to the knockdown of pathogenic peptides/proteins and overexpression of curative circRNAs encoding peptides/proteins, the third important strategy is to use these peptides/proteins along with anticancer drugs. The combination of these peptides/proteins with other anticancer drugs can be directly delivered to the cancer cells *via* exosome or nanoparticle *via* injecting into the patients as anticancer therapy [**Figure 4C(iii)**].

Despite the huge volume of publications on circRNA involvement in tumorigenesis, the underlying mechanisms are still not completely clear. Exploring the hidden mechanisms of circRNAs and circRNA-encoded peptides will recognize their importance in human cancers (128, 129). In the future, more studies on the development of effective technologies to target circRNAs *in vivo* will lead to the clinical revolution of circRNA-based therapeutics. In this regard, circRNAs databases play an important role and provide valuable information; improvements in these databases and other detection technologies will provide new strategies for cancer treatment. Some circRNAs databases are summarized here in **Table 2**.

**Table 2:** circRNAs databases.

Name	Website	Source of data	Description	Reference
CircBase	http://www.circbase.org/	*Homo sapiens*, *Mus musculus*, *C. elegans*, *Drosophila melanogaster*, and *Latimeria chalumnae*	CircBase contains genomic locations, sequences, and gene descriptions	(130)
CIRCpedia v2	http://www.picb.ac.cn/rnomics/circpedia	Human, mouse, rat, zebrafish, fly, and Worm	Contains an bioinformatics tool for comparison of the differential expression of circRNAs and conservation	(131)
CircInteractome	http://circinteractome.nia.nih.gov	CircInteractome searches public circRNA, miRNA, and RBP databases and provide analyses of binding sites on circRNAs and analyzes miRNA and RBP sites on junction and flanking sequences	To find out binding sites on circRNAs, genomic and mature circRNA sequences, potential circRNAs act as RBP sponges, junction-spanning primers for circRNAs, siRNAs for circRNA silencing and to identify IRES	(132)
CircNet database	http://circnet.mbc.nctu.edu.tw/	CircNet constructed by transcriptome sequencing datasets	Provide circRNAs, integrated miRNA and gene regulatory networks; expression profiles, genomic annotations, sequences of circRNA isoforms, and tissue-specific circRNA expression profiles	(133)
CirclncRNAnet	http://app.cgu.edu.tw/circlnc	An integrated web-based resource for functional networks of lncRNAs and circRNAs	Provide a regulatory networks of lncRNAs and circRNAs of interest based on NGS-based expression data by cross-referencing databases	(134)
CircRNADb	http://reprod.njmu.edu.cn/circrnadb	A comprehensive database for human circular RNAs with protein-coding annotations	Provide information of the circRNA, includes protein encoding annotation, exon splicing, genome sequence, IRES, ORF and references	(47)
CircR2Disease	http://bioinfo.snnu.edu.cn/CircR2Disease/	Database for experimentally supported circRNAs associated with various diseases	Provide circRNA expression information and mechanisms and find the algorithms for circRNA disease associations	(135)
Tissue-specific circRNA database (TSCD)	http://gb.whu.edu.cn/TSCD	Tissue-specific circRNAs of humans and mice	Identify functions of tissue-specific circRNAs and explore new biomarkers for development of disorders	(136)
Cancer-specific circRNA database (CSCD)	http://gb.whu.edu.cn/CSCD	An integrated interactional database of cancer-specific circRNAs	Aim to identify cancer-specific circRNAs and the functional effects of circRNAs	(137)
exoRBase	https://www.exoRBase.org	A database of circRNA, lncRNA, and mRNA derived from RNA-seq data analyses of human blood exosomes	The integration and visualization of RNA expression profiles based on RNA-seq data of both normal individuals and patients with different diseases	(126)
MiOncoCirc	https://mioncocirc.github.io/	The database composed of circRNAs detected in tumor tissues	MiOncoCirc, will be bank for the circRNA function, exploration, and development of circRNA as a diagnostic or therapeutic biomarker in cancers	(138)
CircAtlas 2.0	https://circatlas.biols.ac.cn/	The integration of over one million circRNAs generated a atlas from six different species; human, macaca, mouse, rat, pig, chicken	A functional circRNA resource to identify circRNAs expression profile, conservation, and functional annotation	(139)
CircBank	https://www.circbank.cn/index.html	A comprehensive database for human circRNA including more than 140,000 human annotated circRNA from different source	To identify miRNA binding site, conservation, m^6^A modification, mutation, and protein-coding potential of circRNAs	(140)
NoncoRNA	https://www.ncdtcdb.cn:8080/NoncoRNA/	A database of experimentally supported ncRNA and drug target associations in cancer	Provide a resource to explore drug-sensitive/resistance ncRNAs in various human cancers	(141)
DeepBase v2.0	http://biocenter.sysu.edu.cn/deepBase/	DeepBase v2.0 collects >18,000 circRNAs in human (>92.5%), mouse, an d*C. elegans* from deep sequencing data	Provide the comprehensive expression profiles. functions and evolutional patterns of diverse ncRNAs	(142)
Circ2Traits	http://gyanxet-beta.com/circdb/	This integrates 1,953 circRNAs related to human diseases, their genomic annotation, and miRNA-binding sites. The included circRNA contains disease-related SNPs or interaction with disease-related miRNAs	Provide the knowledge of potential association of circRNAs with diseases in human and putative miRNA–circRNA–mRNA–lncRNA interaction network for each diseases	(143)

## Conclusion and Future Prospective

CircRNAs are a highly stable class of ncRNAs and are directly involved in gene-cell cycle regulation that makes them flashy targets for cancer studies. circRNAs and their encoding peptides/proteins take part in fundamental cellular processes like gene expression, cell proliferation, and migration of cancer cells, which are considered as the hallmark of cancer development through suppressing or promoting the different cancers either in differential peptides or in miRNA sponges. However, this bifunctionality of circRNAs needs further in-depth exploration.

The current assessment of the literature on circRNAs demonstrates an expanding body of data supporting the effectiveness of circRNAs encoding peptides/proteins in clinical practice for different malignancies. The current research established a significant relationship between the ectopic expression of circRNA-encoded peptides and prognostic as well as diagnostic values in oncology. For their effective involvement in cell cycle, drug resistance, and functioning in protein translation, circRNAs provide a new avenue of clinical research to identify new peptide-based cancer biomarkers for diagnosis and potential use to generate circRNA-peptide-based targeted therapeutic drugs.

Although ongoing research on circRNA-encoded peptides’ role in progression and as diagnostic targets for different medical illnesses including cancer holds great promise, especially in terms of precision medicines and to design multi-marker approaches toward cancer diagnosis and treatments, it is still in its early stages of exploration. Summarizing the research on classification, biogenesis, peptide translation, and the clinical applications of circRNAs, we come to conclude with some prospect points here.

Firstly, only a handful of functional circRNA-encoded peptides/proteins have been characterized yet. We endorse more extensive studies on the biology of circRNAs especially about protein-coding ability and their interactions with gene expression will be launched. We also endorse to develop an academic standardized nomenclature system for circRNAs. Secondly, except as miRNA sponges, it is imperative to elucidate other possible mechanisms underlying circRNAs participating in regulation of gene expression. Third, there is a need to implement large multiethnic studies before recommending specific circRNA-encoded peptides/proteins for related cancer diagnosis or treatment. Fourth, for the ultimate goal of using circRNAs as a potential target for cancer diagnoses or therapy, future research should be directed to deal with host immune rejection and delivery of circRNA-encoded peptides/proteins to the particular sites *in vitro*. Fifth, the discovery of dysregulated circRNA-encoded peptides/proteins should be mainly carried out in clinical tissue samples. In the future, the expression of circRNA-encoded peptides/proteins should be discovered in more clinical samples associated with a specific disease, such as blood, urine, saliva, breast milk, and other body fluids.

We anticipate that the coming days’ in-depth new research will strengthen the applications to develop circRNA-encoded peptide-based diagnostic and therapeutic approaches. This review could promote the blooming growth of research on circRNAs and their clinical application in the cancer clinics.

## Author Contributions

FAK write the first draft, refined, edited, and revised the manuscript, designed the tables and figures. NHK, BN, SZ, EEN, YW formatted and contributed to the materials organization and final editing of the manuscript. WZ and SJ conceptualized and designed the study. All authors listed have made a substantial, direct and intellectual contribution to the work and approved for publication.

## Funding

This work was supported by the National Natural Science Foundation of China (No. 31371386 SP.J).

## Conflict of Interest

The authors declare that the research was conducted in the absence of any commercial or financial relationships that could be construed as a potential conflict of interest.

## Publisher’s Note

All claims expressed in this article are solely those of the authors and do not necessarily represent those of their affiliated organizations, or those of the publisher, the editors and the reviewers. Any product that may be evaluated in this article, or claim that may be made by its manufacturer, is not guaranteed or endorsed by the publisher.

## Glossary

**Table:**

aa	amino acid
ACC1	acetyl-coa carboxylase 1
ADRA1	adenosine deaminase acting on RNA
AGO2	argonauterisc catalytic component 2
AKT3	AKT serine/threonine kinase 3
ALU	arthrobacter luteus
AMPK	amp-activated kinase
ankrd52	ankyrin repeat domain 52
ASO	antisense oligo
BC	breast cancer
BIRC6	baculoviral IAP repeat-containing 6
Ccnb1	g2/mitotic-specific cyclin-b1
CC	colon cancer
Cdr1	cerebellar degeneration-related *protein*1
CD19	cluster of differentiation 19
ceRNA	competent endogenous rna
Cdk1	cyclin-dependent kinase 1
CDR1as	cerebellar degeneration-related protein 1
CDDP	*cisplatin* (cancer *drug*)
CD44	cluster of differentiation 44
circRNA	circular RNA
ciRNA	circular intronic RNA
ci-sirt7	sirtuin 7
ciRS-7	cerebellar degeneration-related *protein* 1
CircLgr4	leucine-rich repeat-containing g-protein coupled receptor 4
circPPP1R12A	protein phosphatase 1 regulatory subunit 12a
c-Myc	cellular myelocytomatosis
CpG	5′-c-phosphate-g-3′
CSCs	cancer stem cells
DNA	deoxyribonucleic acid
*DNMT1*	DNA methyltransferase 1
e-cadherin	epidermal phenotype protein e
ecircRNA	exonic circular RNA
EIciRNA	exonic–intronic circular RNA
EIF3J	eukaryotic translation initiation factor 3 subunit j
EIF4A3	eukaryotic translation initiation factor 4a3
EGFR	epidermal growth factor receptor
EMT	epithelial–mesenchymal transition
E7	human papillomavirus e7 oncoprotein
FBXW7	f-box and wd repeat domain containing 7
FECR1	*fli1*exonic circular rna
FLI1	friend leukemia virus integration
FNDC3B	fibronectin type III domain containing 3b
FOXO3	forkhead box protein o3
FOXP4	forkhead box p4
FZD	frizzled receptors
GC	gastric cancer
GFP	green fluorescent protein
Gprc5a	g protein-coupled receptor class c group 5 member a
GPCR	g protein-coupled receptor
GSK3β	glycogen synthase kinase 3 beta
H2AX	h2a histone family member x
HCC	hepatocellular carcinoma
HIPK3	homeodomain interacting protein kinase 3
HRCR	heart-related circular RNA
HPV	human papillomaviruses
HuR	human antigen r
ICSs	intronic complementary sequences
IHC	immunohistochemistry
IL-2	interleukin-2
IRESs	internal ribosome entry sites
IRS2	insulin receptor substrate 2
ITCH	itchy e3 ubiquitin protein ligase
kDa	kilodalton
lncRNA	long non-coding RNA
m^6^A	N6-methyladenosine
MBL/MBLN1	muscle blind
mcm5	minichromosome maintenance protein 5
miRNA	micro RNA
MRE	micro RNA response element
MTO1	mitochondrial transfer RNA translation optimization 1
N-cadherin	mesenchymal phenotype n
ncRNA	non-coding RNA
nt	nucleotide
ORF	open reading frame
PABPN1	poly(a) binding protein nuclear 1
PAF1	polymerase-associated factor
PAIP2	poly(a) binding protein interacting protein 2
PDK1	pyruvate dehydrogenase kinase isozyme 1
PI3K/Akt	phosphatidylinositol 3-kinase/akt serine/threonine kinase
LINC-PINT	long intergenic non-protein coding RNA, P53 induced transcript
piRNA	piwi-interacting RNA
Pol II	polymerase-II
Poly (A)	poly adenine
p53	protein p53
p70S6K	p70 ribosomal S6 kinase
QKI	quaking
RBPs	RNA-binding proteins
RCA	rolling circle amplification
RNA	ribonucleic acid
rRNA	ribosomal RNA
RRL	rabbit reticulocyte lysate
SHPRH	SNF2 histone linker PHD RING helicase
siRNA	small interfering RNA
SNCA	synuclein alpha
snoRNA	small nucleolar RNA
snRNA	small nuclear RNA
SOCS3	suppressor of cytokine signaling 3
sry	sex region y
Sp1	specificity protein 1
TADA2A	transcriptional adaptor 2A
TET1	ten-eleven translocation methylcytosine dioxygenase 1
TNBC	triple negative breast cancer
TNF-α	tumor necrosis factor alpha
tRNA	transfer RNA
U2AF65	u2 auxiliary factor65
UPF1	up-frameshift-1
USP28	ubiquitin-specific protease 28
UTR	untranslated region
Wnt/β-catenin	wingless and int-1/β-catenin
WT	wild type
YAP	yes-associated protein
ZNF91	zinc finger protein 91
ZNF609	zinc finger protein 609
